# Heat Shock Cognate 70 Functions as A Chaperone for the Stability of Kinetochore Protein CENP-N in Holocentric Insect Silkworms

**DOI:** 10.3390/ijms20235823

**Published:** 2019-11-20

**Authors:** Bingqian Li, Zhiqing Li, Chenchen Lu, Li Chang, Dongchao Zhao, Guanwang Shen, Takahiro Kusakabe, Qingyou Xia, Ping Zhao

**Affiliations:** 1 Biological Science Research Center, Southwest University, Chongqing 400715, China; libingqian@swu.edu.cn (B.L.); luchenchenlcc@163.com (C.L.); cl17783721226@163.com (L.C.); zdc6666@163.com (D.Z.); gwshen@swu.edu.cn (G.S.); xiaqy@swu.edu.cn (Q.X.); zhaop@swu.edu.cn (P.Z.); 2 Chongqing Key Laboratory of Sericultural Science, Chongqing Engineering and Technology Research Center for Novel Silk Materials, Southwest University, Chongqing 400715, China; 3 Laboratory of Insect Genome Science, Kyushu University Graduate School of Bioresource and Bioenvironmental Sciences, Fukuoka 819-0395, Japan; kusakabe@agr.kyushu-u.ac.jp

**Keywords:** *Bombyx mori*, centromere, kinetochore, CENP-N

## Abstract

The centromere, in which kinetochore proteins are assembled, plays an important role in the accurate congression and segregation of chromosomes during cell mitosis. Although the function of the centromere and kinetochore is conserved from monocentric to holocentric, the DNA sequences of the centromere and components of the kinetochore are varied among different species. Given the lack of core centromere protein A (CENP-A) and CENP-C in the lepidopteran silkworm *Bombyx mori*, which possesses holocentric chromosomes, here we investigated the role of CENP-N, another important member of the centromere protein family essential for kinetochore assembly. For the first time, cellular localization and RNA interference against CENP-N have confirmed its kinetochore function in silkworms. To gain further insights into the regulation of CENP-N in the centromere, we analyzed the affinity-purified complex of CENP-N by mass spectrometry and identified 142 interacting proteins. Among these factors, we found that the chaperone protein heat shock cognate 70 (HSC70) is able to regulate the stability of CENP-N by prohibiting ubiquitin–proteasome pathway, indicating that HSC70 could control cell cycle-regulated degradation of CENP-N at centromeres. Altogether, the present work will provide a novel clue to understand the regulatory mechanism for the kinetochore activity of CENP-N during the cell cycle.

## 1. Introduction

The faithful transmission of genetic information among generations of cells requires the accurate congression and segregation of chromosomes during mitosis [1,2,3]. An important role in this process is performed by the centromere, a specialized chromatin region in the chromosome marked by a specific histone H3 variant CenH3, also named centromere protein A (CENP-A), instead of the canonical histone H3 [4,5,6,7]. During mitosis, CENP-A will recruit kinetochore proteins to centromeres so as to establish the connection between chromosomal DNA and microtubules of the mitotic spindle [8,9,10].

In eukaryotes, there are two kinds of centromere chromosomes—monocentric and holocentric [11]. For monocentric chromosomes, the paired chromosomes are joined at a single point or primary constriction [12]. In contrast, centromeres in holocentric species are distributed along extensive segments or even the entire length of the paired chromosomes [13]. Although the function of the centromere and kinetochore is conserved from monocentric to holocentric, the lack of conservation for DNA sequences of the centromere and components of the kinetochore among different species indicates the complexity of kinetochore assembly on the centromere [14,15,16].

CenH3 or CENP-A has been widely accepted as an essential marker for centromere specification in most eukaryotic species [5,17]. The loss of CenH3 homologs in several holocentric insects, however, challenges the concept and may indicate a novel way to define the feature of centromeres [18]. In addition to the diverse centromeres, more than 30 core kinetochore proteins have been identified in either monocentric or holocentric species to date [19]. They are subdivided into two layers according to their centromere positions; one is the centromere-proximal layer, also called inner kinetochore proteins, which are the constitutive centromere associated network proteins (CCAN), and the other layer is the microtubule-proximal layer, also known as outer kinetochore proteins, which contain proteins of the so-called KMN network including Knl1, Mis12, and Ndc80 complexes [20,21]. The recent findings have shown that the kinetochore proteins are varied in different species even in the same monocentric type. For example, at least 16 inner kinetochore proteins are identified in humans [10,12], whereas only CENP-C was successfully identified in *Drosophila melanogaster* [22].

The domesticated silkworm *Bombyx mori* has proven to be an excellent lepidopteran model organism for studying genetics and genomics and has been reported to possess holocentric chromosomes [23,24]. Except for *Caenorhabditis elegans* whose holocentromeres have been well studied [25,26,27], the features of centromeres and components of kinetochores in other holocentric species remain largely unknown. Based on the complete genomic information of the silkworm, previous work has identified several inner and outer kinetochore proteins, including eight putative CCAN proteins (CENP-E, -I, -K, -L, -M, -N, -S, and -X) and four KMN subunits (Ndc80, Mis12, Dsn1, and Nnf1) [18,28,29]. The functions of these kinetochore proteins or unknown components in centromere formation, however, still need to be explored.

Among the kinetochores, CENP-C and CENP-N have been reported to be involved in the recognition of CENP-A nucleosome position through interaction with CENP-A and are required for kinetochore assembly and chromosome segregation [30,31,32]. It has been shown that the central region and the CENP-C motif in CENP-C are essential for interacting with the C-terminal tail of CENP-A and thus targeting itself to the centromere [30]. As for CENP-N, the N-terminal region is critical for binding to the L1 loop of CENP-A and this binding is stabilized by electrostatic interactions with the nucleosomal DNA [32,33]. On the other hand, the C-terminal region of CENP-N is confirmed to be responsible for interacting with the other CCAN proteins via CENP-L [34].

Due to the lack of CENP-A and CENP-C in silkworms [18], in this work, we sought to investigate the role of CENP-N in cell division and discover the proteins that interact with CENP-N. The functional exploration of kinetochore proteins in silkworms should be useful for further comparative analysis with other holocentric species. Cellular localization and RNA interference (RNAi) silencing of CENP-N studies in silkworm cells have confirmed its kinetochore functions. An affinity purification–mass spectrometry approach was used to identify the interactions and we obtained 142 factors that were specifically enriched in the CENP-N complex. Among the factors, it was interestingly found that heat shock cognate 70 (HSC70), a molecular chaperone, is able to interact with CENP-N and the depletion of HSC70 leads to decreased expression of CENP-N. Therefore, we concluded that HSC70 plays a critical role in regulating the stability of kinetochore protein CENP-N in silkworms.

## 2. Results

### 2.1. Kinetochore Function of CENP-N in Silkworms

**Figure 1:**
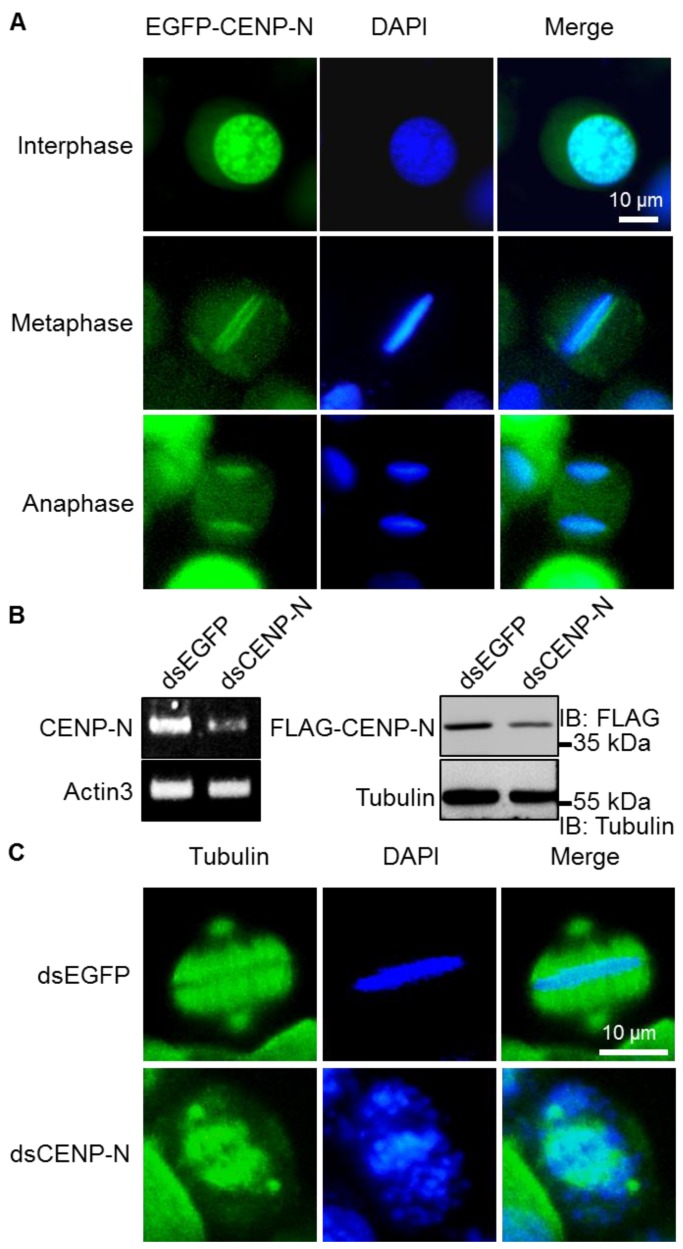
Kinetochore function of CENP-N in silkworms. (**A**) Representative images of silkworm cells expressing EGFP-CENP-N in different cell cycle phases. CENP-N was labeled with EGFP fluorescence (green) and cell cycle phases were determined by DAPI (blue). Scale bar, 10 μm. (**B**) RT-PCR and Western blotting assays of RNAi efficiency for CENP-N in cultured silkworm BmN4-SID1 cells stably expressing FLAG-CENP-N. The cells were treated with control dsRNA or CENP-N dsRNA, and the expression of actin3 and tubulin were used as loading controls, respectively. (**C**) Representative immunofluorescence images of mitotic phenotypes following CENP-N knockdown. Cells were fixed and stained with anti-tubulin antibody (red) and the nuclear DNA were stained with DAPI (blue). At least 10 metaphase cells were recorded for CENP-N knockdown. Scale bar, 10 μm.

In order to investigate the function of kinetochore proteins in the holocentric insect silkworm, we first cloned the CENP-N homologous gene from the cDNA library of cultured silkworm BmN4-SID1 cells. Consistent with the previous reports, EGFP-tagged CENP-N was primary localized in the nucleus at interphase, and clearly formed dot signals at the both sides of chromosome DNA at metaphase (Figure 1A), which exhibited the expected kinetochore localization [35].

To analyze the role of CENP-N during the cell cycle, we performed RNAi experiments on CENP-N in cultured silkworm BmN4-SID1 cells. Upon dsRNA-mediated RNAi, RT-PCR and Western blotting analysis exhibited that both transcriptional and translational levels of CENP-N were significantly decreased (Figure 1B), which showed the efficient RNAi for CENP-N in cells. When we examined cell mitosis after CENP-N RNAi, it was clearly shown that knockdown of CENP-N significantly induced deficient congression and segregation of chromosomes at metaphase (Figure 1C). These observations thus confirmed that CENP-N is a functional kinetochore component in silkworms.

### 2.2. Identification of the CENP-N Complex

**Figure 2:**
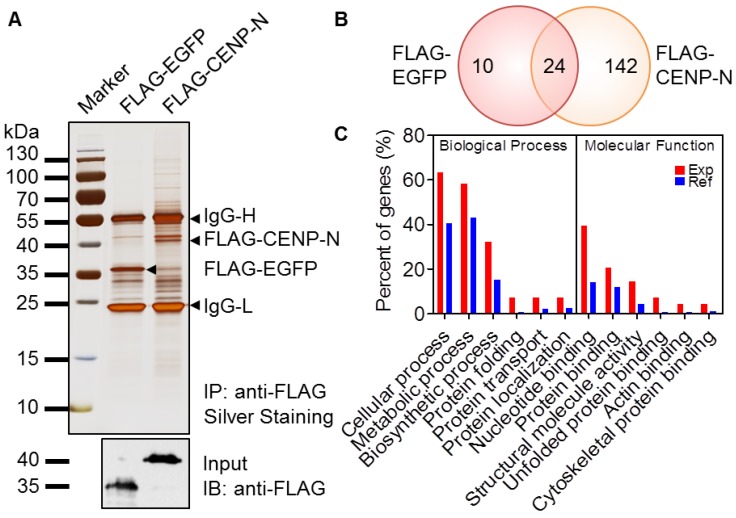
Identification of the CENP-N complex. (**A**) Silver stained SDS-PAGE gel of anti-FLAG affinity purifications from the indicated stable cell lines. Arrowheads indicated the target proteins and heavy/light chains of IgG. Input proteins were also used to detect the target proteins by Western blotting. (**B**) Venn diagram assay of the potential CENP-N interacting proteins identified by LC-MS/MS. (**C**) Functional annotation of the CENP-N interacting proteins as experiment group (Exp) was analyzed by WEGO, and the total silkworm genes as reference group (Ref) were also shown in the figure. Proteins were classified into molecular function and biological process according to their GO signatures.

To identify the potential centromeric proteins in silkworms, we established a silkworm BmN4-SID1 cell line stably expressing a FLAG-tagged CENP-N protein. After the collection of solubilized proteins from cells, anti-FLAG affinity purification was performed to isolate the interacting proteins of CENP-N. As a control, FLAG-tagged EGFP expressing cells were used for a similar analysis. Based on the Western blotting result, the two cell lines could effectively express the targeted proteins, respectively (Figure 2A). After affinity purification, silver staining showed that FLAG-CENP-N was able to pull down many more proteins compared to the FLAG-EGFP control (Figure 2A).

To further identify the proteins in the eluate, we performed LC-MS/MS analysis. As a result, we obtained 142 specific proteins that have potential interactions with CENP-N after removing the overlapping proteins in the control treatment (Figure 2B and Supplementary Table S2). To confirm the interaction of CENP-N with the identified proteins, we selected four candidates including cyclin dependent kinase (CDK)10, FCPa, FCPb, and HSC70, which are associated with cell cycle progression. For instance, the smooth progression and completion of the cell cycle is dependent on a series of positive and negative regulatory factors, such as CDK10 [36]. FCP proteins play important roles in regulating the number and length of microfilaments and may also be involved in extracellular signaling, cytoskeletal reorganization, and motor behavior [37]. HSC70 protein is involved in the regulation of cell division, molecular chaperone activity, signal transduction, and transcription and translational control [38]. As shown in Supplementary Figure S1, co-localization and co-IP experiments have revealed the interactions between CENP-N and target proteins which validate the LC-MS/MS data.

To understand the functions of the identified proteins, we carried out a Blast2GO analysis to annotate protein functions using the WEGO program. It was shown that these proteins could participate in various cellular processes and mainly possess binding activities with nucleotides and proteins (Figure 2C). These analyses thus suggested that the interacting proteins with CENP-N may contribute to its kinetochore function.

### 2.3. Reduced Expression of CENP-N by HSC70 Depletion

**Figure 3:**
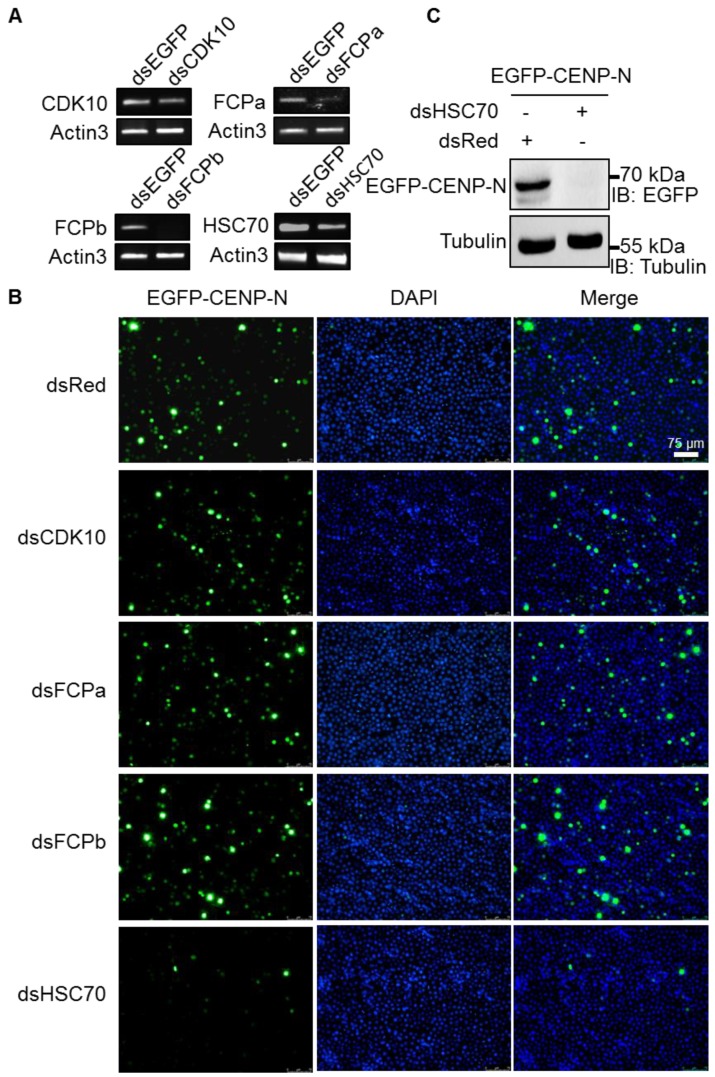
Reduced expression of CENP-N by heat shock cognate 70 (HSC70) depletion. (**A**) Knockdown efficiency of cyclin dependent kinase (CDK)10, FCPa, FCPb, and HSC70 was evaluated by RT-PCR. The expression of the actin3 gene was used as an internal control. (**B**) Effects of CDK10, FCPa, FCPb, and HSC70 knockdown on the expression of EGFP-CENP-N. EGFP-CENP-N stably expressing cells was knocked down by dsRNA for each gene. CENP-N was labeled with EGFP fluorescence (green) and the nuclear DNA were stained with DAPI (blue). Scale bar, 75 μm. (**C**) Knockdown of HSC70 decreased the expression of EGFP-CENP-N by using an anti-EGFP antibody. The expression of tubulin was used as an internal control. “+” and “-” represented the addition and non-addition of designated dsRNA, respectively.

To investigate the effect of interacting proteins on CENP-N, we synthesized dsRNAs against target genes (CDK10, FCPa, FCPb, and HSC70) and conducted RNAi experiments in BmN4-SID1 cells stably expressing the EGFP-CENP-N protein. After treating the cells with dsRNAs, RT-PCR showed that the transcriptional levels for each gene were strongly attenuated by the specific dsRNAs (Figure 3A). It was interesting that knockdown of HSC70 significantly reduced the fluorescent signals of EGFP-CENP-N, which was not observed for CDK10, FCPa, FCPb, or control Red gene (Figure 3B). Moreover, Western blotting analysis also showed the decreased expression of EGFP-CENP-N by using an anti-EGFP antibody, which further confirmed the observation (Figure 3C). Owing to the molecular chaperone activity of HSC70, we can speculate that HSC70 would be specifically required for the stability of CENP-N.

### 2.4. Stability of CENP-N via Interaction with HSC70 Chaperone

**Figure 4:**
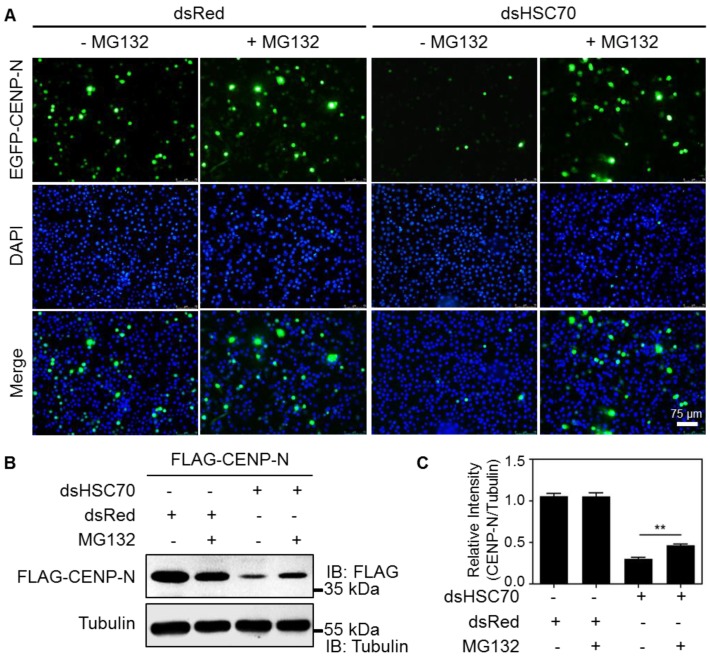
Stability of CENP-N via interaction with HSC70 chaperone. (**A**) Representative images of silkworm cells expressing EGFP-CENP-N under different treatments. CENP-N was labeled with EGFP fluorescence (green) and the nuclear DNA were stained with DAPI (blue). Scale bar, 75 μm. (**B**) Representative immunoblotting images of silkworm cells expressing FLAG-CENP-N under different treatments. CENP-N was detected using an anti-FLAG antibody and the expression of tubulin was used as an internal control. “+” represented the addition of designated dsRNA and MG132, and “-” represented the non-addition of dsRNA and MG132. (**C**) Quantification of FLAG-CENP-N expression levels using Image J software. “+” represented the addition of designated dsRNA and MG132, and “-” represented the non-addition of dsRNA and MG132. Three independent replicates were analyzed and statistical significance was evaluated by GraphPad Prism 5 (** *p* < 0.01).

The stability of CENP-N may be largely dependent on the ubiquitylation of the protein itself. We next hypothesized that the HSC70 chaperone is involved in the protection of CENP-N degradation by the ubiquitin–proteasome proteolytic system. To test this hypothesis, we used the proteasome inhibitor MG132 to treat cells. As shown in Figure 4A, the HSC70 depleted cells without MG132 treatment greatly reduced the expression signals of EGFP-CENP-N, whereas the fluorescent signals were significantly restored in the presence of MG132. To confirm this observation, the cells stably expressing FLAG-CENP-N were also treated at the same way. Western blotting using an anti-FLAG antibody showed a similar result—in response to treatment with MG132, the expression of FLAG-CENP-N was enhanced in cells without HSC70 (Figure 4B,C). These data revealed that HSC70 functions as a chaperone for the stability of CENP-N so as to prohibit the proteasomal degradation of kinetochore proteins.

## 3. Discussion

The histone H3 variant CenH3 or CENP-A is an essential feature of centromeres, which provides a unique structure to assemble kinetochore proteins [5,7]. However, genome-wide identification of CenH3 homologs in several insect orders has shown a significant difference [18]. For instance, insects belonging to dipteran, hymenopteran, and coleopteran orders possess CenH3 homologs, whereas lepidopteran, hemipteran, and phthirapteran insects do not [18]. It is interesting that the chromosomes of insects with the CenH3 protein are monocentric while the insects without CenH3 have holocentric chromosomes [18]. These specific profiles show the critical role of CenH3 as a marker in monocentric insects but not the indispensable role of CenH3 in holocentric insects, which may suggest a CenH3-indenpendant kinetochore assembly mechanism in holocentric insects. Therefore, the identification of novel kinetochore proteins and functional analysis of known kinetochore proteins in holocentric species will be crucial for understanding the mechanism of kinetochore assembly in holocentromeres.

Given the important connection of CENP-N in the inner kinetochore layer with CENP-A and CENP-C [31,32,33], in the present work, we investigated the function of CENP-N, which has been previously identified to be present in the lepidopteran silkworm that possesses holocentric chromosomes, but not CENP-A and CENP-C homologs [29]. For the first time, we confirmed the kinetochore function of CENP-N in cultured silkworm cells and performed affinity purification–MS to identify CENP-N interacting proteins. Although we did not find the known kinetochore proteins such as CENP-L, CENP-I, and CENP-M that are able to form a complex with CENP-N in mammalian kinetochore structures [34,39,40], we did identify specific proteins including cell cycle-associated factors that could interact with CENP-N. The missing identification of reported kinetochore proteins in the CENP-N complex in silkworms may be due to the dynamic interaction of kinetochore proteins during the cell cycle and very severe purification conditions done in the present work. Among of these proteins, we further provided evidence that the molecular chaperone HSC70 is involved in the stability of CENP-N through the ubiquitin–proteasome pathway.

CENP-N plays an essential role in cell cycle progression and cell mitosis [33,41]. Localization of CENP-N in different cell phases and incorrect congression and segregation of chromosomes by depletion of CENP-N in silkworms have uncovered its kinetochore function. During the cell cycle, it has been shown that constant mRNA levels of CENP-N are maintained whereas the protein levels in different phases vary in humans [42]. For instance, CENP-N will accumulate in the S phase and decrease following cell cycle progression, which means that the CENP-N protein will specifically undergo degradation in mitosis. However, how to regulate the degradation of CENP-N during the cell cycle remains unclear.

Molecular chaperones and their accessory proteins are capable of activating and folding proteins, playing important roles in the ubiquitin–proteasome pathway, and are able to maintain protein stability. Indeed, it has been shown that the chaperone protein HSP90 can regulate the E3 ligase activity of the CUL4A complex, which in turn contributes to CENP-A ubiquitylation and CENP-A deposition at the centromeres [43]. Our present data exhibited that another chaperone protein, HSC70, was able to interact with CENP-N and that knockdown of HSC70 significantly decreased the expression of CENP-N. Moreover, the CENP-N levels were recovered by treatment with a proteasome inhibitor, MG132. All these data thus revealed that the chaperone protein HSC70 may control cell cycle-regulated degradation of CENP-N at centromeres. The specific cell phase in which HSC70 prohibits the degradation of CENP-N during the cell cycle, however, still needs to be elucidated.

It has long been a research hotspot to decipher the characterization of centromeres in various species, because centromeres differ greatly in sequence organization among species [44]. A prevalent view is that the tandem repeat sequences are highly conserved at centromeres of both animal and plant genomes [45,46]. Centromere tandem repeats at centromeres, however, lack conserved sequence properties. Therefore, identifying the sequence property in the holocentric silkworm genome is important for understanding centromere functions. The loss of CENP-A homologs in silkworms made it difficult to identify the centromere locations in the genome [18]. The present evidence on the kinetochore function of CENP-N in silkworms has thus provided new clues about mapping the sequences at centromeres through chromatin immunoprecitation sequencing (ChIP-seq) with an antibody against the CENP-N protein. Our stably expressing FLAG-CENP-N cells would also provide a promising approach to identify the genome-wide localization of centromeres in silkworms using a reliable FLAG antibody.

## 4. Materials and Methods

### 4.1. Plasmids

Expression constructs for CENP-N, CDK10, FCPa, FCPb, and HSC70 were amplified from the cDNA library of cultured silkworm cells using the primers (BGI, Shenzhen, China) listed in Table S1. These genes were inserted into a NcoI–XhoI or NcoI–NotI site of the pENTR11 (Invitrogen, Carlsbad, CA, USA) vector [47]. All plasmids were verified by sequencing. The pENTR11 for CENP-N was further cloned into the expression vectors of pPBO_ie2GW (containing the N-terminal EGFP tag) and pPBO_ie2FW (containing the N-terminal FLAG tag) by gateway reaction to construct the expression plasmids of EGFP-CENP-N and FLAG-CENP-N [47,48]. The pENTR11 clones of CDK10, FCPa, FCPb, and HSC70 were inserted into pPBO_ie2RW (containing the N-terminal Red tag) and pPBO_ie2HW (containing the N-terminal HA tag) vectors in the same way to construct their expression plasmids, respectively.

### 4.2. Cell Culture and Transfection

In this study, we used the cultured silkworm ovary-derived BmN4-SID1 cell line, which has been widely used for efficient RNA interference experiments in silkworms [49]. The BmN4-SID1 cell line was maintained at 27 °C in IPL-41 medium (Gibco, Waltham, MA, USA) supplemented with 10% fetal bovine serum (Hyclone, Logan, UT, USA). The expression plasmids for EGFP-CENP-N and FLAG-CENP-N were inserted into the genome of BmN4-SID1 cells using the piggyBac transposition system according to the previous report [48], and the stably transformed cells were selected by resistance to puromycin (CalBiochem, Darmstadt, Germany). For transient transfections, various plasmids were transfected into cells using X-tremeGENE HP DNA transfection reagent (Roche, Basel, Switzerland) according to the manufacturer’s instructions.

### 4.3. RNA Interference

The synthesis of double stranded RNAs (dsRNAs) for CENP-N, CDK10, FCPa, FCPb, and HSC70 were carried out by T7 RNA polymerase in vitro. The dsRNAs for the control genes EGFP and Red were also synthesized. RNA interference (RNAi) experiments were conducted in BmN4-SID1 cells and RNAi efficiency for each gene was assayed by RT-PCR according to our previous studies [50,51]. Briefly, the BmN4 SID-1 cells were previously cultured in 24-well or 6-well plates at a cell density of 0.5 × 10^5^ or 2.0 × 10^5^, respectively, in IPL-41 medium with 10% FBS. Each dsRNA for EGFP, Red, CENP-N, CDK10, FCPa, FCPb, and HSC70 was added to the medium at a final concentration of 0.5 μg/mL. Five days after incubation with dsRNAs, the cells were harvested to extract RNA and conduct RT-PCR assays.

### 4.4. Immunoblotting

Cells were homogenized in IP lysis buffer (P0013, Beyotime, Shanghai, China) containing a mixture of protease inhibitors (Beyotime, Shanghai, China). The supernatants were collected by centrifugation at 14,000 rpm at 4 °C for 10 min. The resulting solutions were mixed with an equal volume of 2× SDS sample buffer, boiled, and resolved by 12% SDS-PAGE. The immunoblotting assay was performed with the following antibodies: anti-FLAG (AF519, Beyotime, Shanghai, China), anti-HA (AH158, Beyotime, Shanghai, China), anti-GFP (ab290, Abcam, Cambridge, UK) [52]. Signals were detected with Thermo Fisher Scientific ECL reagent (32106, Pierce, Waltham, MA, USA) under a ChemiScope (CLiNX, Shanghai, China) Western blot processor.

### 4.5. Immunofluorescence

For subcellular localization analysis, cells were cultured on a cover slip, fixed with 3.7% formaldehyde in phosphate-buffered saline (PBS) for 10 min, and the nuclear DNA were stained by 4’,6-diamidino-2-phenylindole (DAPI) (Invitrogen, Carlsbad, CA, USA). For microtubule spindle analysis, BmN4-SID1 cells after RNAi of CENP-N or EGFP were incubated with an anti-tubulin monoclonal antibody (ab7291, Abcam, Cambridge, UK) to label the spindle microtubules, and the second antibody was FITC-labeled goat anti-mouse IgG (A0568, Beyotime, Shanghai, China) [52,53]. Fluorescent images were captured by using a Leica microscope (Z16, Leica, Wetzlar, Germany).

### 4.6. Immunoprecipitation

For the co-immunoprecipitation (Co-IP) assay, cells co-expressing the targeted proteins were harvested using IP lysis buffer (P0013, Beyotime, Shanghai, China) containing a mixture of protease inhibitors (Beyotime, Shanghai, China). The lysates were immunoprecipitated using an anti-HA antibody (AH158, Beyotime, Shanghai, China), and the eluted proteins were further detected by Western blotting using anti-FLAG (AF519, Beyotime, Shanghai, China) and anti-HA (AH158, Beyotime, Shanghai, China) antibodies [52]. For purification of the CENP-N complex, cells stably expressing FLAG-CENP-N were collected and lysed in RIPA buffer (50 mM, Tris-HCl (pH 7.5), 150 mM NaCl, 2 mM EDTA, 1% NP-40, and 0.25% sodium deoxycholate) with protease inhibitors. The cell supernatants were further incubated with anti-FLAG affinity gel beads (Sigma, Saint Louis, MO, USA) and purified according to the previous protocol [50]. Briefly, the affinity gel beads were washed with RIPA buffer five times, and then eluted by incubation with 200 μL elution buffer (50 mM Tris-HCl (pH 7.5), 50 mM DTT, 1 mM EDTA, 1% SDS, and 10% glycerol). The eluates were collected for Western blotting and liquid chromatography–tandem mass spectrometry (LC-MS/MS) assays.

### 4.7. Ubiquitin–Proteasome Inhibitor Assay

The BmN4-SID1 cell line, stably expressing FLAG-CENP-N or EGFP-CENP-N, was cultured in 24-well or 12-well plates, and dsRNA for Red or HSC70 were added, respectively. After three days of RNAi treatment, the proteasome inhibitor MG132 (Millipore, Darmstadt, Germany) was added at a final concentration of 10 µM for 12 h. Cells were collected for fluorescent observation and immunoblotting detection.

### 4.8. Silver Staining

After separation of proteins by SDS-PAGE, the gel was fixed in the fixative solution (25 mL methanol, 6 mL glacial acetic acid, 25 μL formaldehyde, and 19 mL Milli-Q H_2_O) for 1 h, and washed in the cleaning solution (25 mL methanol, and 25 mL Milli-Q H_2_O) for 30 min. After the fixer reaction (0.015 g NaS_2_O_3_, and 50 mL Milli-Q H_2_O) for 5 min and staining solution (0.1 g AgNO_3_, 25 μL formaldehyde, and 50 mL Milli-Q H_2_O) for 30 min, the gel was developed in the solution (0.005 g NaS_2_O_3_, 3 g Na_2_CO_3_, 25 μL formaldehyde, and 50 mL Milli-Q H_2_O).

### 4.9. LC-MS/MS Assay

After digestion of the purified CENP-N complex, LC-MS/MS was performed to identify the protein components according to the previous protocol [50]. Briefly, the protein solution after immunoprecipitation was chemically reduced with 10 mM DTT for 1 h at 37 °C, and then alkylated with 50 mM iodoacetamide for 1 h at room temperature in the dark. After washing with 8 M urea and 50 mM NH4HCO3 in an ultrafiltration tube, proteins were digested with trypsin for 20 h at 37 °C. The peptide mixture was acidified by 0.1% formic acid and resolved by using a Thermo Fisher Scientific EASY-nLC 1000 system (Waltham, MA, USA) under the standard parameters.

### 4.10. Data Analysis

For protein identification, the raw data were analyzed with MaxQuant software (version 1.3.0.1, https://www.maxquant.org/) against an integrated silkworm proteome database according to the published procedure [54]. At least one unique peptide was designated as an identified protein and the protein information was listed in Table S2. After removing the common proteins presented in the FLAG-EGFP, only the proteins present in FLAG-CENP-N were identified as interacting proteins with CENP-N. For protein annotation in the CENP-N complex, we used the Blast2GO program (https://www.blast2go.com/) [55] to search against the non-redundant protein database (NR, NCBI, https://www.ncbi.nlm.nih.gov/). The WEGO database (http://wego.genomics.org.cn/) was used to analyze the interacting proteins.

## 5. Conclusions

By combining functional RNAi experiments and LC-MS/MS analysis, we have investigated the kinetochore function of CENP-N and identified its interacting proteins in silkworms. Importantly, we provided the first evidence that the chaperone protein HSC70 is able to regulate the stability of CENP-N. Future work should focus on deciphering the mechanism of how HSC70 is involved in the protection of CENP-N degradation during the cell cycle, and analyzing the sequence properties of holocentric chromosomes in silkworms.

## Supplementary Materials

Supplementary materials can be found at https://www.mdpi.com/1422-0067/20/23/5823/s1.
