# Supplementary figures and images for: Heat Shock Cognate 70 Functions as A Chaperone for the Stability of Kinetochore Protein CENP-N in Holocentric Insect Silkworms
Source: Int J Mol Sci. 2019 Nov 20;20(23):5823. doi: 10.3390/ijms20235823 (PMC6929194; doi:10.3390/ijms20235823)

## Supplementary Figure S1

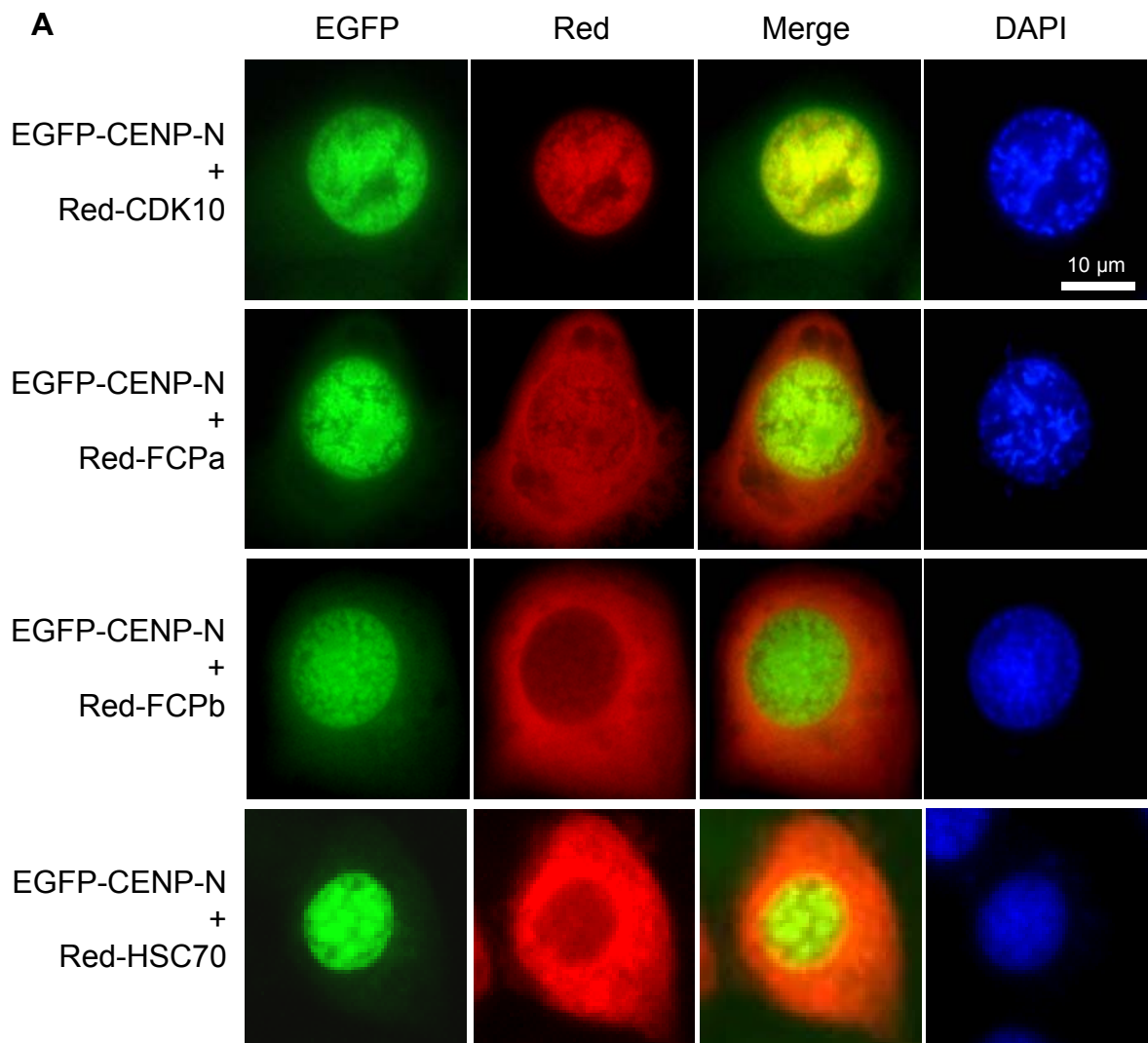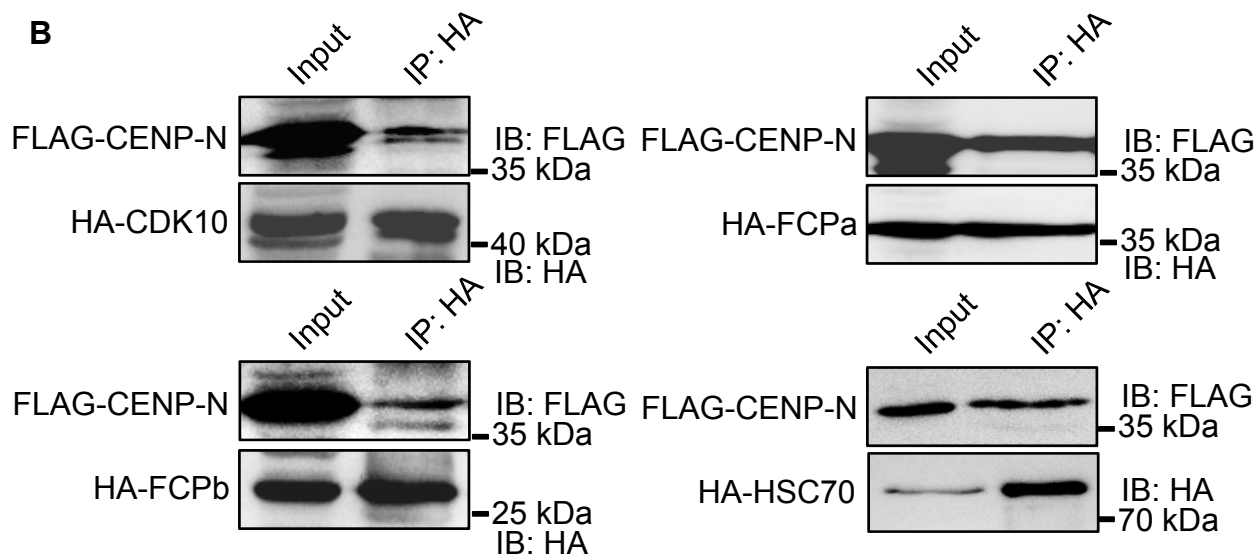

Supplement: Supplementary file 1 [file ijms-20-05823-s001.zip › Figure S1.pdf]
